# Impact of the COVID-19 pandemic on hernia surgery in a Swedish healthcare region: a population-based cohort study

**DOI:** 10.1186/s12893-022-01698-6

**Published:** 2022-07-05

**Authors:** Christos Kollatos, Sarmad Hanna, Gabriel Sandblom

**Affiliations:** 1 Department of Surgery, Ryhov County Hospital, 55185 Jönköping, Sweden; 2 Department of Surgery, Kalmar Hospital, Kalmar, Sweden; 3 Department of Clinical Science and Education, Södersjukhuset, Karolinska Institute, Stockholm, Sweden; 4 Department of Surgery, Södersjukhuset, Stockholm, Sweden

**Keywords:** Covid-19, Hernia, Emergency, Elective surgery, Incarceration

## Abstract

**Background:**

Swedish healthcare has been reorganised during the COVID-19 pandemic, affecting the availability of surgery for benign conditions. The aim of this study was to determine the effects of COVID-19 on emergency and elective hernia surgery in a Swedish healthcare region.

**Methods:**

Using procedure codes, data from inguinal and ventral hernia procedures performed at the three hospitals in Jönköping Region, Sweden, from March 1st 2019 to January 31st 2021, were retrieved from a medical database. The cohort was divided into two groups: the COVID-19 group (March 1st 2020–January 31st 2021) and the control group (March 1st 2019–January 31st 2020). Demographic and preoperative data, hernia type, perioperative findings, and type of surgery were analysed.

**Results:**

A total 1329 patients underwent hernia surgery during the study period; 579 were operated during the COVID-19 period and 750 during the control period. The number of emergency ventral hernia repairs increased during the COVID-19 period, but no difference in inguinal hernia repair rate was seen. The characteristics of patients that underwent hernia repair were similar in the two groups. Moreover, the decrease in elective ventral hernia repair rate during the COVID-19 period did not result in a higher risk for strangulation.

**Conclusion:**

There is no evidence to suggest that the decrease in the number of elective ventral hernia repairs during the COVID-19 period had any impact on the risk for strangulation. Indications for surgery in patients with a symptomatic ventral or inguinal hernia should be carefully evaluated. Studies with greater power and longer follow-up are needed to gain a full understanding of the effects of the COVID-19 pandemic on hernia surgery.

## Introduction

The global pandemic of coronavirus disease 2019 (COVID-19) has had far-reaching effects in Sweden [1]. The virus SARS-CoV-2, that can cause severe acute respiratory syndrome, was first confirmed in Sweden on the 31st January 2020 when a woman returning from Wuhan tested positive. Unlike many other Western countries, Sweden has not imposed a lockdown and has kept a large part of society open [2].

The COVID-19 pandemic has had a great impact on the healthcare system, forcing widespread reallocation of healthcare personnel [3, 4]. This has led to the postponing of numerous planned procedures for benign conditions [5–7].

On the other hand, it has provided a unique opportunity to study the impact of watchful waiting on the outcome of symptomatic hernias, since repairs have been postponed due to the pandemic. To date, few studies have focused specifically on watchful waiting as a way of managing symptomatic hernias [8–11], and most of these have been carried out during the COVID-19 pandemic [12]. Lima et al. described a fall in emergency hernia surgery rate during a pandemic [13]. Similar findings were found in the German Hernia Register [14]. Moreover, there was no change in mortality rates following abdominal wall hernia repairs during the COVID-19 pandemic despite an increase in emergency procedures [15].

In this study, we investigated the impact of the COVID-19 pandemic on hernia surgery in a Swedish healthcare region where no lockdown measures were taken. The hypothesis was that the rate of emergency surgery increased during the COVID-19 pandemic leading to higher morbidity rates.

## Materials and methods

### Study design

This was a population-based cohort study. Collection of data was performed according to STROBES statement for observational studies [16]. Data were obtained from the digital medical records in Jönköping Region, with a catchment of approximately 360.000 inhabitants. There are three hospitals in Jönköping Region all of which perform emergency surgery. Planned hernia repairs, however, are only performed in two of them. There is little private healthcare and no private unit performs emergency surgery.

Patients who had undergone hernia repair between 1st March 2019 and 31st January 2021, were included. The patients were divided into two groups; the control group (1st March 2019–31st January 2020) and the COVID-19 group (1st March 2020–31st January 2021). These timeframes were chosen because 1st March 2020 was the date the first wave of COVID-19 began and by 31st January 2021 the second wave had ended, allowing the healthcare system to at least temporarily return to normal.

All hernia repairs were identified by the Swedish Procedure Coding System [17]. Hernia repairs on children under 18-years-of-age were excluded.

The variables analysed were demographic data (age, sex), date and type of surgery, anaesthetic risk according to American Society of Anesthesiologists (ASA score), hernia type, use of mesh, and medical procedure codes for procedures undertaken at the same time.

In cases where patients were admitted with an incarcerated inguinal or ventral hernia, the first line treatment was to attempt manual reduction of incarcerated tissues from the hernia sack to the natural compartment. If the contents were reduced, repair was usually undertaken within 48 h. If the hernia could not be reduced, the hernia was repaired without greater delay than was necessary to stabilise the patient and access the necessary resources (staff, operating theatre). Whatever, all such repairs were registered as emergency procedures.

### Statistical analysis

Descriptive statistics and statistical analyses were performed using IBM SPSS Statistics 27.0.1.0. Chi-square test and Fisher’s exact test were used to compare categorical variables between groups. Student t-test was used to analyse continuous variables. A p-value < 0.05 was considered statistically significant.

### Ethics

The study was approved by the Swedish Ethics Review Body (Ref. no. 2022-00061-01).

## Results

During the study period, 1329 hernia repairs were identified. Altogether 579 hernia repairs were performed in the COVID-19 group. Of these, 68 (12%) were carried out as an emergency procedure and 511 (88%) as a planned procedure. During the control period, there were 750 hernia procedures, 672 (90%) performed as a planned procedure and 78 (10%) as an emergency procedure (Table 1). There was no significant increase in emergency hernia repair rate during the COVID-19 period (p = 0.44).

**Table 1:** Emergency and elective hernia repairs from March 2020 to January 2021 (COVID-19 group) and March 2019 to January 2020 (Control group)

	Inguinal hernia repair			Ventral abdominal wall hernia			Total hernia repair		
	Emergency	Elective	*p* 0.34	Emergency	Elective	*p* 0.01	Emergency	Elective	*p* 0.44
COVID-19	26 (6%)	389 (94%)		42 (26%)	122 (74%)		68 (12%)	511 (88%)	
Control group	40 (8%)	467 (92%)		38 (16%)	205 (84%)		78 (10%)	672 (90%)	

Only 6% of all inguinal hernia repairs were carried out as an emergency procedure, but emergency procedures were performed in 22% and 34% of primary ventral hernias and incisional hernias respectively (Table 1). Furthermore, when comparing the number of operations for each type of hernia in the COVID-19 and control groups, an increase in emergency ventral abdominal wall hernia repair rate was seen during the COVID-19 period (26% vs. 16%, *p* = 0.01). The greatest increase was seen for incisional hernia repairs with more than double the number of repairs performed as an emergency procedure in the COVID-19 group (34% vs. 15%, *p* = 0.02, Table 2).

**Table 2:** Emergency and elective ventral hernia repairs from March 2020 to January 2021 (COVID-19 group) and March 2019 to January 2020 (Control group)

Primary ventral hernia repair			Incisional hernia repair		
Emergency	Elective	*p* 0.16	Emergency	Elective	*p* 0.02
26 (22%)	91 (78%)		16 (34%)	31 (66%)	
28 (16%)	150 (84%)		10 (15%)	55 (85%)	

As expected, numerous elective operations were postponed during the COVID-19 pandemic, with 16% and 40% decreases in the number of inguinal and ventral hernia repairs respectively. In all, the number of elective hernia repairs during the pandemic fell to 161.

**Fig. 1:**
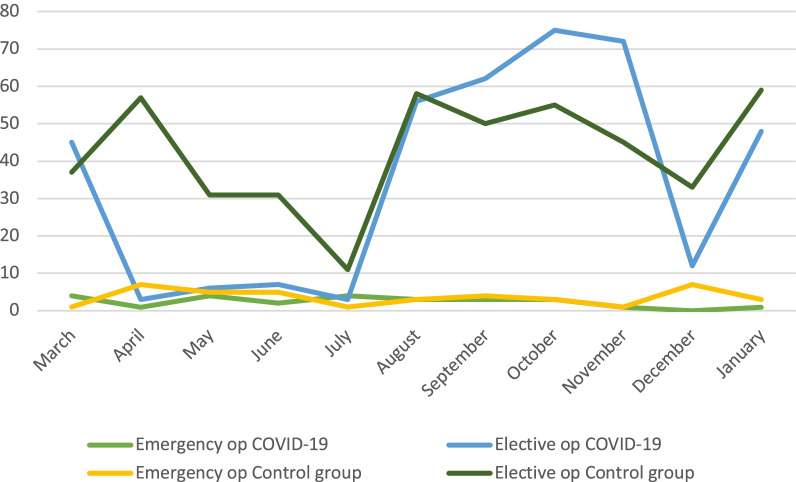
Emergency and elective inguinal hernia operations March 2020–January 2021 vs. March 2019–January 2020. ‘Crisis situation’ agreement was activated between August and November 2020

**Fig. 2:**
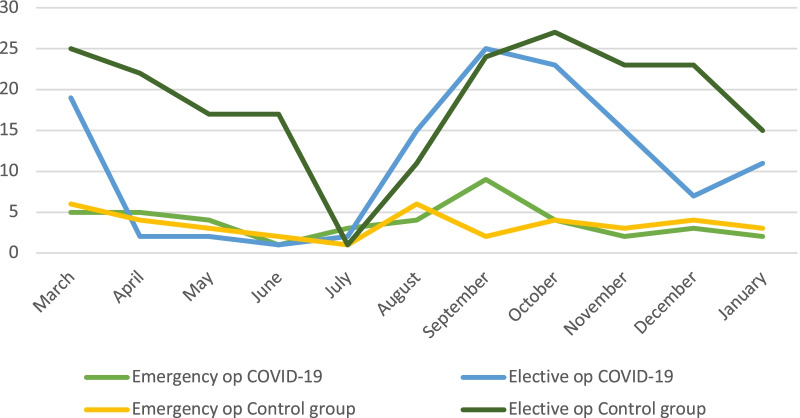
Emergency and elective ventral hernia operations March 2020–January 2021 vs. March 2019–January 2020. ‘Crisis situation’ agreement was activated between August and November 2020

In August 2020, a crisis situation agreement was reached in Jönköping Region, where staff were obliged to prolong working hours. This provided the opportunity to perform more elective operations and lasted until November 2020. Initially, day-case surgery was given priority, but even the number of postponed major ventral hernia repairs was significantly less than the period between March and July 2020 (Figs. 1, 2).

Comparative analysis did not show any differences between the two groups, indicating that the characteristics (age, sex, ASA score) of patients that underwent a hernia repair were similar in both groups (Table 3). The reduction in elective hernia repair did not result in a higher risk for strangulation or organ resection (Table 4). The same pattern was seen for emergency ventral hernia repair, where differences were not statistically significant (*p* = 0.12).

**Table 3:** Demographic and preoperative characteristics of patients who underwent emergency hernia repair

	COVID-19	Control group	
Male	37	51	*p* 0.24
Female	30	28	
Age, years (mean)	60	65	*p* 0.16
ASA 1	12	15	*p* 0.12
ASA 2	28	31	
ASA 3	23	27	
ASA 4	5	5

**Table 4:** Emergency hernia repairs complicated by organ resection in the COVID-19 and control groups

	COVID-19	Control group	
Inguinal	3	2	*p* 0.052
Ventral	3	0	
Incisional	1	0	

## Discussion

The overall number of emergency hernia repairs did not increase during the COVID-19 period in the Jönköping healthcare region, despite the decrease in planned procedures. There was, however, a moderate increase in the number of emergency repairs for incisional hernia. This indicates that the volume of planned hernia repairs on the population-level has relatively little impact as a preventive measure against unexpected incarceration. The increase in emergency incisional hernia repairs may be explained by the greater risk for incarceration in incisional hernias compared to primary ventral and inguinal hernias.

During the first phase of the pandemic i.e., between March and July 2020, only emergency hernia surgery was performed. Elective hernia repair was limited to less than 10 and 5 operations per month for inguinal and ventral hernia respectively compared to the previous year, with only few exceptions. However, after the crisis situation agreement in August 2020, previously postponed procedures were carried out. In particular, day-care surgery for inguinal hernia and minor ventral hernia repairs was given priority. This explains the large fluctuations in ventral hernia repairs and especially incisional hernia repairs during the COVID-19 period. Furthermore, the COVID-19 pandemic did not have any impact on the number of inguinal hernia repairs as almost all of these operations are carried as day-case surgery in our region.

The decrease in elective ventral hernia repair rate was followed by a moderate increase in emergency repairs for incisional hernia. This study shows a 64% increase in emergency ventral hernia surgery when elective cases fell by 40%. It is important to mention here that patients that are planned for hernia surgery in Jönköping Region must have a symptomatic hernia, whilst watchful wait is applied in cases with asymptomatic hernia. Moreover, no change in surgical approach was seen during the COVID-19 pandemic because the local routines of open or laparoscopic approach remained unchanged.

There was no statistically significant increase in the number of cases with incarceration or strangulation requiring organ resection. This must be interpreted with some caution since the study lacks statistical power to detect minor increases in risk. We cannot, therefore, conclude that it is generally safe to postpone planned hernia repairs. There are potential confounders affecting the decision to carry out emergency surgery, but there were no differences between the two groups regarding patient characteristics. A limitation of our study is that no data about BMI, previous surgery or comorbidity index were included. ASA score as a measure of anesthesiology risk was the only variable patient-related risk factor analyzed.

The retrospective data analyses and small sample size limit the validity of the study. The crisis situation agreement had a major impact on the pattern of hernia surgery in Jönköping Region, especially on major ventral hernia surgery. These results are derived from a Swedish Region with specific pandemic measures and, therefore, they may thus not be generalizable to countries that have met the Covid-19 pandemic with other healthcare measures [18, 19]. However, we believe that a low elective ventral hernia repair rate has a negatively affect due to the higher risk for emergency operations and therefore risk for organ resection.

## Conclusion

In conclusion, no increase in the total number of emergency inguinal hernia repairs was seen in the COVID-19 group, however a significant increase of more than 60% in the number of emergency ventral hernia repairs. This increase was associated with a 40% reduction in elective ventral hernia repairs. The number of emergency procedures where organ resection was necessary remained unchanged. The crisis situation agreement had a great impact on the number of patients in the region operated for hernia, and we believe that hernia repair may be performed during the pandemic when the necessary precautions have been taken. These results suggest that indications for surgery in patients with a symptomatic ventral or inguinal hernia should be carefully evaluated, but that planned surgery has only limited potential to reduce the rate of emergency hernia surgery.

## Data Availability

The datasets used and analyzed during the current study are available from the corresponding author on reasonable request.
